# The Nature, Pathology and Treatment of Dipsomania

**Published:** 1881-11-20

**Authors:** Edward C. Mann

**Affiliations:** New York; Physician to Sunnyside, a Private Hospital for the Treatment of Dipsomania, the Opium Habit and Nervous and Mental Diseases; Office 28 West 50th Street


### Southern Medical Record:

EDITORS:

T. S. Powell, M.D. W. T. Goldsmith, M.D. R. C. Word, M.D.

*R. C. WORD, M.D., Managing Editor.*

☞All Communications and Letters on Business connected with the Record must be addressed to the Managing Editor.

Vol. XI. ATLANTA, GA., NOVEMBER 20, 1881. No. 11.

### ORIGINAL AND SELECTED ARTICLES.

### THE NATURE, PATHOLOGY AND TREATMENT OF DIPSOMANIA.

BY EDWARD C. MANN, M. D., OF NEW YORK.

Physician to Sunnyside, a Private Hospital for the Treatment of Dipsomania, the Opium Habit and Nervous and Mental Diseases.

(*Concluded.*)

PATHOLOGY OF INEBRIETY.

The basis of our cerebral pathology is the fundamental principle, that healthy mental function is dependent upon the proper nutrition, stimulation and repose of the brain; and upon the processes of waste and reparation being regularly and properly maintained. We know that the cerebral cells are nourished by the proper and due supply of nutritive plasma from the blood, and that this is essential to healthy function; and, indeed, the ultimate condition of the mind, with which we are now acquainted, consists in the due nutrition, growth and renovation of the brain-cells. If now, we take into the system an amount of alcohol that causes the blood-plasma to carry to the brain-cells a noxious and poisonous, in place of a nutritive substance, stimulating the cells so as to hasten the progress of decay and waste beyond the power of reparation and renovation, and impressing a pathological state in them, we must inevitably have resulting a change in healthy function and a certain amount of disease induced. Owing to the abuse of alcohol, we have resulting a change in the chemical composition of the cerebral cells from the standard of health, which is the foundation of organic disease, as it prevents and interrupts healthy function. As a result of the overfilling of the cerebral vessels or cerebral hyperæmia from the long-continued use of alcohol, we have at first, symptoms of *irritation*, due to increased excitability of the nerve-filaments and ganglion cells of the brain. The symptoms of *exhaustion* and *depression* occurring at a later stage are due to lost excitability of the nerve-filaments and ganglion cells of the brain, owing to a want of the proper supply of arterial oxygenated blood to them. This is caused by the excessive cerebral hyperæmia, the escape of venous blood from the brain being obstructed, the result being that no new arterial blood can enter the capillaries. We may have apoplectiform or epileptiform attacks and paralysis occurring in the course of these cerebral hyperæmias, and they may be due either to obstructed escape of venous blood or to secondary œdema of the brain, in which transudation of serum takes place into the perivascular spaces and interstitial tissue of the brain with consequent anæmia. We know comparatively little yet respecting the physiology and pathology of the nervous system, and consequently comparatively little information has been gained regarding the morbid changes that take place in the brain and its appendages as a result of the abuse of alcohol. Such knowledge as we do possess shows that analogous changes take place in chronic alcoholism and chronic insanity, namely, atrophy and induration of the brain, and thickening and infiltration of the membranes.

The nerve-cells have also been found to be the seat of granulation in some instances, and some histologists have claimed to have discovered fatty degeneration of the various brain elements. Respecting the latter changes, Dr. J. Batty Tuke, of Edinburgh, who is one of the most successful of modern investigators in the department of morbid cerebral histology, gives it as his opinion that the application of the various tests for oil will fail to detect the presence of the so-called "free oil globules" in the substance of the convolutions, which he considers to be but the scattered debris of granular cells. According to the great pathologist, Rokitansky, we find thickening and increase of the pia-mater and arachnoid and permanent infiltration of the former and a varicose condition of its vessels as a result of continued abuse of alcohol. As the state of the pia-mater is unquestionably closely related to the higher functions of the brain, the latter must suffer more or less as the result of such an abnormal condition of the former. If there exists a permanently congealed and thickened state of the pia-mater, it is extremely probable that if it becomes suddenly turgid and hyperæmic as a result of severe emotional disturbances, we shall have, resulting from the increased pressure on the brain, coma, epileptiform and apoplectiform attacks, and other grave nervous symptoms.

It is fair to conclude that in the majority of cases the first changes that occur are repeated attacks of active cerebral congestion, followed by chronic cerebral congestion and chronic cerebral meningitis; and that as the disease assumes a chronic form the brain takes on a secondary change and becomes anæmic, atrophied and indurated—a state allied to cirrhosis. In these cases of chronic meningitis, proceeding to atrophy and induration—of which I have seen quite a number—the prominent symptoms have been impairment of memory, dullness of intellect bordering on dementia, trembling of the limbs, tottering gait, hesitating, slurring speech, and other symptoms indicative of gradually progressing paralysis. In two cases of general paralysis, due to drink, in which I made a *post mortem* examination, paying careful attention to the state of the brain and spinal cord, I found in both instances thickening and opacity of the membranes with adherence to each other and to the brain, showing the existence of chronic meningitis. The brain was, in both instances, anæmic and indurated, and in one case there was dilatation of the lateral ventricles with considerable effusion. The spinal cord was atrophied and indurated, and there was considerable fluid in the spinal cord in one of the cases and also at the base of the brain. Upon hardening the spinal cord and making thin sections, and employing carmine staining to demonstrate the structural relation of the cord more clearly, I found, upon microscopical examination, that there was atrophy and loss of nerve elements of the posterior columns, with a new formation of connective tissue.

In making autopsies where the cause of death has been owing, directly or indirectly, to the abuse of alcohol, I have found cirrhosis of the liver, fatty and waxy liver, cancer of the liver, chronic Bright's disease, cancer of the stomach and cancer of the bladder; and in one case, a gummy tumor of the dura-mater. It is doubtless true that in many cases we shall find, upon examination, no pathological changes in the brain, that are demonstrable by existing knowledge and appliances; but I think we should rather doubt the quality of our resources of observation than doubt the existence of pathological changes in this most delicate, sensitive and complex of all organs, when we have observed during life its functions to be obviously perverted, if not destroyed.

*Treatment of Dipsomania*—Having endeavored to prove that dipsomania is a physical disease—that it is in fact a distinct type of mental disorder, I pass, in conclusion, to the consideration of the question of the cure of this disease. Dipsomania, if properly treated, is curable, as other diseases are. In the treatment of dipsomania we have primarily to build up and restore shattered constitutions and broken-down nervous systems. We have a class of patients to deal with whose digestive powers are weakened; whose appetite is impaired; whose muscular system is enfeebled, and whose generative function is often decayed. The blood is impoverished and the general nutrition disordered. These patients are indirectly predisposed to the acquisition of nearly all diseases, as they have, by long indulgence in alcohol, lessened the power of resisting their causes. We have to deal with the results of a toxic poison which has resulted in more or less pathological change in the brain and nervous centers. We have also to deal, at times, with various complications proceeding from the abuse of alcohol, such as cirrhosis of the liver, gastritis, epilepsy, various forms of dyspepsia, and in some cases with Bright's disease. We must place our patient under the most favorable hygienic influences, provide for him cheerful, tranquil and pleasant surroundings, repress cerebral excitement, procure sleep for him, and we must also give him plenty of good, nourishing food and abundance of fresh air and exercise.

In the treatment of the nervous exhaustion and premature mental decay, we should primarily direct our attention to the direction of the moral habits. We should endeavor to provide constantly easy and pleasant occupation of the mind. We have in these cases to deal with a worn, irritable condition of the nervous system; an unstable condition, as regards its nutrition, its solidity, and its perfection of structure, which makes our task no easy matter. We must be very careful that we make our patients sleep, or we shall have a preponderance of waste over repair that will balk all our efforts. A warm bath of half an hours' duration with a cold towel on the head, with a dose of 15 grains chloral hydrate, and 5 to 15 minims of the fluid extract of hyoscyamus or 30 grains of sodium bromide with 30 minims of the tincture of canabis indica together act well as cerebral sedatives, and promote sleep. It is necessary to supply the greatest amount of nutritive material to the brain and nervous system to repair the undoubtedly existing nutritive lesion. We must quiet all abnormal nervous excitability, and keep our patients calm and tranquil. Attention should be paid to maintaining an even temperature of the body. Care should be paid to the excretory functions of the skin, kidneys and bowels. If there is headache and drowsiness, such diuretics as the liq. ammoniæ acetat. with spt. nitric ether are indicated. Indian hemp has also proved itself in my hands a valuable adjunct in doses of 1/4 gr. of the extract as required. In combination with the bromides it makes one of the best nerve sedatives, or it may be combined with lupulin. Free exposure without fatigue cannot be too strongly insisted on. One of the most valuable remedial agents is phosphorus, which I always prescribe, to be administered in cod-liver oil in doses of from 1-100 to 1-12 of a grain after meals. The cod-liver oil is one of the best nutritive remedies, as fat *must* be applied to the nutrition of the nervous system, if this is to be maintained in its organic integrity. It always has seemed to me that cod-liver oil exercised a specific action in all the hereditary diseases, and it certainly does much good in dipsomnia, which is strongly hereditary. The general effects of phosphorus are those of a stimulant, but it possesses a special power over the exhausted nervous system. It is a nerve food. It is, possibly, evanescent in its effects, but is never followed by a stage of depression which is noticeable. Fairchild's phosphorized elixir of calasaya bark, prepared in New York, is a very good way of giving it, as is also Thompson's solution of phosphorus.

The two most valuable nerve tonics in dipsomania are quinine and strychnia. I generally combine the two in a mixture, so that in each teaspoonful there shall be 2 grs. of quinine and 1-60 to 1-32 of a grain of strychnia. The latter exercises a decided antagonistic effect over alcoholism, while patients who take quinine make much better recoveries than those who do not. When there is persistent insomnia, I am accustomed to rely on the use of prolonged warm baths given at bedtime conjoined with my sodium bromide and cannabis mixture, chloral and hyoscyamus combined, or the mono-bromide of camphor—Clin's capsules—in a single dose of 4 grs. Fothergill's solution of hydrobromic acid in doses of from 15 to 40 minims is sometimes useful.

I come finally to speak of the remedial agent which, in my opinion, far surpasses all others in its permanent effects, and which is comparatively little used. I refer to the judicious use of the constant and induced current of electricity. Electricity is a remedial agent which furnishes us with the means of modifying the nutritive condition of parts deeply situated, and of modifying the circulation to a greater extent, I think, than by any known agent. By the judicious employment of the constant or galvanic and induced or faradic currents, we have it in our power to hasten the processes of nerve-growth and nerve-repair, and thereby indirectly hasten the acquisition of nerve-power. The use of electricity does not, I think, act by contributing anything directly to the growth or repair of nerve tissue. Its action, it would seem most probable, is to stimulate and quicken those processes on which the material and functional integrity of the nervous system depends. The action of electricity is always followed, in my practice, by an increase of strength and nerve force, and the results gained are gradual and permanent; while the use of nerve stimulants has always seemed to me to primarily excite the nerve activities proper, and not the nutritive processes upon which the acquisition of power depends. The deceptive results obtained from the use of nerve stimulants depends upon the excitation of nerve activities and the resultant expenditure of nerve power, which is followed by a period of exhaustion varying in degree and duration. The careful and judicious use of electricity has always led, in my hands, to an increase of nervous energy, while the employment of nerve stimulants has appeared to me to lead, in many instances, ultimately to a waste and diminution of nervous energy.

In cases of dipsomania we have abnormal nervous excitability, conjoined with cerebral exhaustion, and the two indications which are urgent are, primarily, for increased rapidity and effectiveness as regards the process of nerve nutritition; and, secondarily, to secure freedom from excitement and diminution of nerve activity, and thereby to check the waste of nerve structure and of power. These indications we can fulfill by the judicious use of electricity and nerve tonics more certainly than by any other means, there being no other such combined sedative, restorative and refreshant to the central nervous system, and we can thus successfully meet all the indications in cases of cerebral exhaustion and threatened mental disease, except that of affording direct nutriment to the brain, which, as I before stated, I endeavor to obtain by rest, cod-liver oil, phosphorus, etc. The use ot electricity seems to supply to the system, in cases of inebriety, the stimulus which has been withdrawn, as my patients have repeatedly told me that while under treatment they experienced little, if any, of the terrible feelings produced by its withdrawal under ordinary circumstances. I have seen this so often that I advance it as a scientific fact, and not as an untested theory. I have had cases of years standing, who have assured me that the application of the electricity has been of more service to them than anything they had previously tried. I have generally employed both currents, the galvanic and faradic; the former as centric galvanization and the latter with the negative electrode at the lower end of the spine, while I apply the positive pole to the cranial centre on the top of the head, the cervical sympathetic, the cilio-spinal centre or region on each side of the seventh cervical vertebra, and up and down the spine, making a seance of fifteen minutes twice daily. I have obtained such excellent results from its use in dipsomania that I hope other gentlemen presiding over institutions similar to mine, may be induced by my success, to give this very important remedy an extended trial, after which I feel sure that they will never willingly relinquish so effective an agent.

Office 28 West 50th Street.

